# Highly Sensitive Amperometric *α*-Ketoglutarate Biosensor Based on Reduced Graphene Oxide-Gold Nanocomposites

**DOI:** 10.1155/2020/4901761

**Published:** 2020-08-01

**Authors:** Gang Peng, Yadong Yu, Xiaojun Chen, He Huang

**Affiliations:** ^1^College of Biotechnology and Pharmaceutical Engineering, Nanjing Tech University, Nanjing 211800, China; ^2^College of Food Engineering, Anhui Science and Technology University, Fengyang 233100, China; ^3^College of Food Science and Pharmaceutical Engineering, Nanjing Normal University, Nanjing 210023, China; ^4^College of Chemistry and Molecular Engineering, Nanjing Tech University, Nanjing 211800, China

## Abstract

Herein, a rapid and highly sensitive amperometric biosensor for the detection of *α*-ketoglutarate (*α*-KG) was constructed via an electrochemical approach, in which the glutamate dehydrogenase (GLUD) was modified on the surface of reduced graphene oxide-gold nanoparticle composite (rGO-Au_nano_ composite). The rGO-Au_nano_ composite was one-step electrodeposited onto glassy carbon electrode (GCE) surface and was characterized by scanning electron microscopy (SEM), energy dispersive X-ray spectroscopy (EDS), and electrochemical techniques. In addition, the rGO-Au_nano_/GCE was also found to electrocatalyze the oxidation of *β*-nicotinamide adenine dinucleotide (NADH) at the peak potential of 0.3 V, which was negatively shifted compared with that at bare GCE or Au_nano_/GCE, illustrating better catalytic performance of rGO-Au_nano_. After the modification of GLUD, the GLUD/rGO-Au_nano_/GCE led to effective amperometric detection of *α*-KG through monitoring the NADH consumption and displayed a linear response in the range of 66.7 and 494.5 *μ*M, with the detection limit of 9.2 *μ*M. Moreover, the prepared GLUD/rGO-Au_nano_/GCE was further evaluated to be highly selective and used to test *α*-KG in human serum samples. The recovery and the RSD values were calculated in the range of 97.9–102.4% and 3.8–4.5%, respectively, showing a great prospect for its real application.

## 1. Introduction

*α*-Ketoglutarate (*α*-KG) is an important metabolic marker of early diagnosis of cancer and microbial fermentation monitoring and also is a vital intermediate in the tricarboxylic acid cycle as well as a node to connect the carbon-nitrogen metabolism in cells [1–4]. It has been emphasized to possess exciting angiogenesis suppressor activity [5–7]. Furthermore, micronutrient application of *α*-KG has exhibited beneficial effects on several malignant tumors with only minor negative effects on normal cells [4,5]. Therefore, accurate and sensitive methods for the detection of *α*-KG are urgently required. Till now, several methods have been developed for the quantification of *α*-KG, such as electrochemical [8], gas chromatography-mass spectrometry (GC-MS) [4], and high-performance liquid chromatography (HPLC) [5, 9]. Among them, electrochemical methods have become considerably meaningful due to the advantages of convenience, high speed, low cost, and easy-to-use. Currently, Wang and his coworkers have developed an electrochemical biosensor for rapid and sensitive detection of *α*-KG based on ruthenium-rhodium modified carbon fiber enzyme microelectrode [8]. However, a large overpotential was found at the microelectrode and the detection of *α*-KG was prone to suffer interference from other compounds. Therefore, it is important to prepare a capable modified electrode with lower overpotential for *α*-KG detection and meanwhile maintain the bioactivity of the immobilized enzyme.

Graphene (Gr) has become extremely attractive in many fields since it has superiorities such as good biocompatibility [10], large surface-to-volume ratio, excellent conductivity [11], electron mobility, and flexibility [12]. In the electrochemical biosensing fields, its large surface-to-volume ratio is helpful for increasing the loading amount of the enzyme, and its excellent conductivity is favourable for transferring electrons between the electrode surface and electrolyte [13–19]. However, the practical applications of Gr are challenged by its irreversible agglomeration or even restack to form graphite due to the Van der Waals interactions in the drying state [20]. Recent research found that noble metal nanoparticles could be used to effectively prevent the massive agglomeration of Gr sheets. Si got a Pt nanoparticles (Pt_nano_)-Gr composite with the Gr partially exfoliated from its drying aqueous dispersions [21]. Tang et al. founded a rapid and efficient one-step approach to prepare Gr-Ag_nano_ composite by simultaneous reduction of Gr oxide (GO) and Ag^+^ with formaldehyde as the reducing agent [22]. Li et al. developed a sensor for the detection of paracetamol based on Pd_nano_-GO composite, which was obtained by a one-pot chemical reduction using Pd^2+^ as a precursor without large aggregation [23]. Also, noble metal nanoparticles-Gr composites have been found to have excellent catalytic performance in electrochemical biosensors [24–26]. Leng et al. demonstrated that the Pd_nano_-GO composite could electrocatalyze the oxidation of rutin, with the detection limit of 0.001 *μ*M [27]. Govindhan et al. prepared Au_nano_-Gr composite which could catalyze NADH with the detection limit as low as 1.13 nM, since the composite provided a large electrochemical active surface area and a favourable environment for electron transfer from NADH to the electrode [28]. Therefore, in this work, we were ready to construct an *α*-KG biosensor based on reduced GO-Au_nano_ (rGO-Au_nano_) composite.

In this paper, the rGO-Au_nano_ composite was one-step electrodeposited onto the glassy carbon electrode (GCE) surface, which exhibited a lower working potential for the electrochemical oxidation of NADH, and served as the platform for the immobilization of glutamate dehydrogenase (GLUD). *α*-KG was detected via monitoring the NADH consumption, and the mechanism was described as in the following equation [8]:

(1) $$\begin{array}{c} \alpha -\text{KG}+{\text{NH}}_{4}^{+}+\text{NADH}\overset{\text{GLUD}}{⟶}L-\text{Glutamate}+{\text{NAD}}^{+}+H_{2}O. \end{array}$$

Catalyzed by GLUD, *α*-KG was conversed to L-glutamate in the presence of NH_4_^+^ and NADH. The more amount of *α*-KG contained in solution, the more NADH was consumed. Then, if a certain amount of NADH was added in advance, the remaining amount of NADH would decrease along with the increase of *α*-KG. Therefore, the concentration of *α*-KG was inversely proportional to the catalytic current of NADH, which provided the quantitative basis for *α*-KG detection.

## 2. Materials and Methods

### 2.1. Materials and Reagents

GO was purchased from Suzhou Hengqiu Co., Ltd. (Suzhou, China). Chloroauric acid and ammonium chloride were obtained from Sinopharm Chemical Reagent Co., Ltd. (Shanghai, China). *β*-nicotinamide adenine dinucleotide reduced dipotassium salt (NADH), L-Glutamic dehydrogenase from bovine liver-Type III (GLUD), and *α*-ketoglutaric acid sodium salt (*α*-KG) were supplied by Sigma Chemical Co., Ltd. (St. Louis, MO, USA). The human serum samples were provided by Jiangsu Center for Clinical Laboratory (JSCCL). All other chemicals were of analytical grade and used without further purification. 0.05 M pH 9.0 of carbonate buffer solution (CBS) and 0.1 M pH 7.2 of phosphate buffered solution (PBS) were used as the electrolyte. Double-distilled water was used throughout the experiments.

### 2.2. Characterization and Electrochemical Measurement

Surface morphologies of rGO and rGO-Au_nano_ films were investigated by scanning electron microscopy (SEM, JEOL, JSM-6510, Japan). The elemental composition analysis was performed by energy dispersive X-ray spectroscopy (EDS, Vantage 4105, NORAN). All electrochemical measurements were carried out on a CHI 650E electrochemical workstation (Shanghai Chenhua Instrument Company, China), employing a typical three-electrode cell system. A modified or bare GCE was utilized as the working electrode (Ф = 3 mm), whereas a saturated calomel electrode (SCE) as a reference and a platinum foil electrode as a counter. All potentials were measured against SCE. All experiments were purged with high-purity nitrogen to remove oxygen and done at room temperature (∼25°C).

### 2.3. Fabrication of *α*-KG Electrochemical Biosensor

**Scheme 1:**
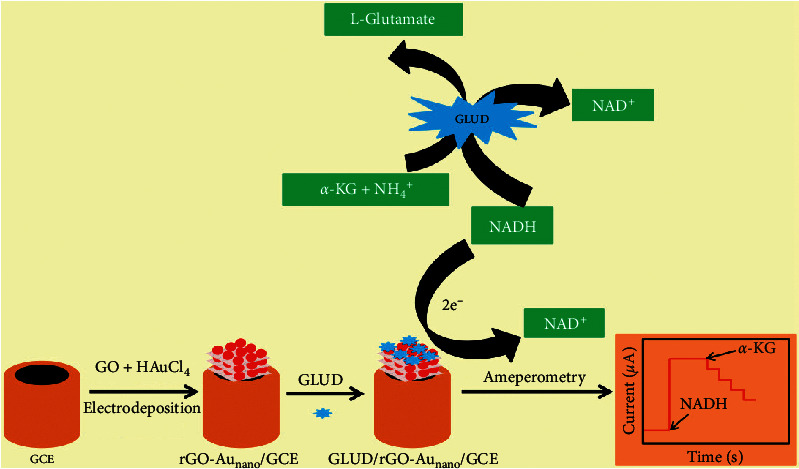
Illustration of the preparation process and the sensing mechanism of the *α*-KG biosensor.

The fabrication process of the electrochemical biosensor was illustrated in Scheme 1. Prior to electrodeposition, the GCE was polished using 0.3 and 0.05 *μ*m alumina powder until a mirror-shiny surface was obtained, and followed by ultrasonication in the ethanol and double-distilled water for 2 min, respectively. The cleaned GCE was then modified by the electrochemical codeposition of the GO and Au_nano_. According to the method reported by Liu et al. [29], GO was exfoliated in CBS by ultrasonication for 20 min to form a homogeneous few-layer GO dispersion. Cyclic voltammetric (CV) reduction was performed in the deposition solutions containing 1.0 mg·mL^−1^ of GO and 200 *μ*M of chloroauric acid with magnetic stirring. The CV was carried out between −1.4 and 0.6 V at a rate of 20 mV·s^−1,^ and the deposition amount was optimized as six potential cycles. Then, 8 *μ*L of 110 kU·L^−1^ GLUD PBS (0.1 M, pH 7.2) was dropped onto rGO-Au_nano_/GCE surface. After natural drying, it was dipped into a 2 wt.% of glutaraldehyde for 3 s to form a protective film and stored in PBS at 4°C before use.

## 3. Results and Discussion

### 3.1. Characterization of rGO-Au_nano_ Composite

**Figure 1:**
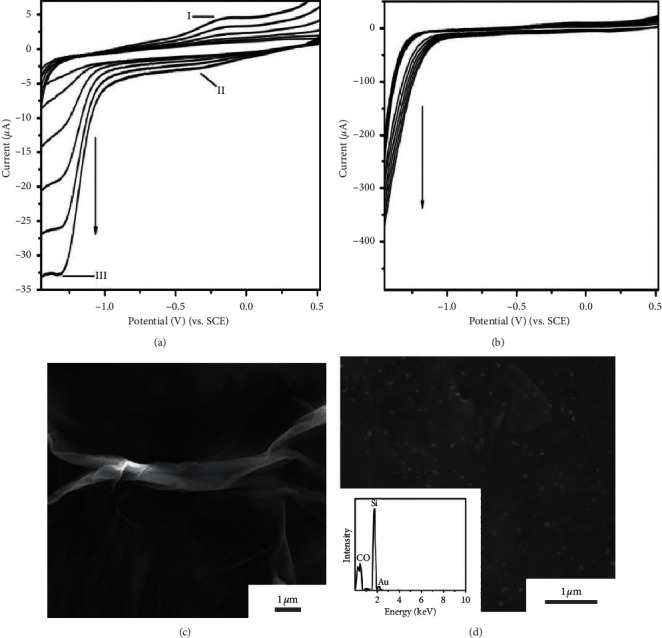
CV for the electrodeposition of (a) 1.0 mg·mL^−1^ GO and (b) 1.0 mg·mL^−1^ GO + 200 *μ*M HAuCl_4_ in 0.05 M pH 9.0 CBS at a scan rate of 20 mV·s^−1^. Typical SEM images obtained from (c) rGO and (d) rGO-Au_nano_ film on Si substrate, respectively. Inset: EDS pattern of the rGO-Au_nano_.

CV was recorded during the electrodeposition of GO on GCE (Figure 1(a)). There is one anodic peak (I) and two cathodic peaks (II and III), corresponding to the redox pair of some oxygen-containing groups [30] and the irreversible electrochemical reduction of GO [31], respectively. The on-going increase of the peak current with successive potential sweeps indicated that the rGO from GO dispersion was successfully deposited onto GCE.  Figure 1(b) shows the CV of codeposition of GO and HAuCl_4_, with a completely different appearance from Figure 1(a). The reduction current was larger than that of GO electrolysis, indicating that the deposition of rGO and Au_nano_ onto the surface of GCE was achieved [29]. The number of electrodeposition cycles was also optimized since the thickness of the rGO-Au_nano_ composite film would influence the conductivity and stability of the rGO-Au_nano_/GCE. This part would be interpreted in detail in the section of the optimization of the experimental parameters.

**Figure 2:**
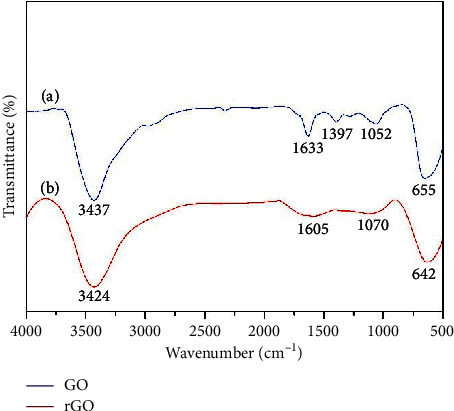
FT-IR spectra of GO (a) and rGO (b).

The morphologies of rGO and rGO-Au_nano_ were characterized by SEM.  Figure 1(c) shows the SEM image of rGO film, in which a typically wrinkled texture was displayed. In Figure 1(d), you could find that Au_nano_ uniformly scattered over rGO to make the rGO-Au_nano_ composite. The Au_nano_ prevented the agglomeration of rGO; meanwhile, rGO enhanced the dispersion of Au_nano_, both of which improve the conductivity and stability of rGO-Au_nano_ composite film. Further, the EDS spectrum confirmed the elemental composition of rGO-Au_nano_, in which Si element comes from the Si substrate. The FT-IR spectra of GO and rGO are shown in Figure 2. The FT-IR spectrum of GO (a) exhibits the characteristic absorptions from oxygen-containing functional groups. In detail, the absorption band at 3437 and 1397 cm^−1^ can be assigned to the stretching vibration and deformation vibration of O-H, respectively. The band at 1052 cm^−1^ belongs to the C-O (alkoxy), while the band at 1633 cm^−1^ derives from the vibration of the adsorbed water molecules and/or the contribution of the skeletal vibration of unoxidized graphitic domains. After the reduction, the peaks at 1070 cm^−1^ assigned to epoxy groups are decreased significantly, clearly indicating the removal of oxygen-containing groups of GO (curve b in Figure 2) and suggesting the successful reduction of GO [32].

### 3.2. Electrochemical Performance of rGO-Au_nano_ Composite

**Figure 3:**
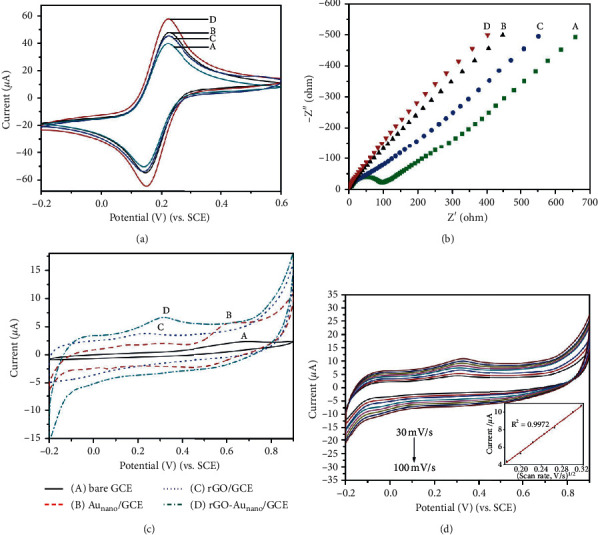
(a) CVs and (b) EIS of the different electrodes: bare GCE (A), rGO/GCE (B), Au_nano_/GCE (C), and rGO-Au_nano_/GCE (D) in 0.1 M KCl containing 5.0 mM [Fe(CN)_6_]^3−/4−^ at a scan rate of 50 mV·s^−1^. (c) CVs of the different working electrode in the presence of 0.5 mM NADH at a scan rate of 50 mV·s^−1^. (d) CVs of rGO-Au_nano_/GCE in 0.5 mM NADH at various scan rates (from inner to outer: 30, 40, 50, 60, 70, 80, 90 and 100 mV·s^−1^). Inset: the plot of anodic peak current against the square root of the scan rate.

Figure 3(a) presents the CVs of four different modified GCEs in 0.1 M KCl containing 5 mM of [Fe(CN)_6_]^3−/4−^ at a scan rate of 50 mV·s^−1^. The current response of rGO/GCE (curve B towards [Fe(CN)_6_]^3−/4−^) was larger than that of bare GCE (curve A), owing to that rGO enhanced the electron transfer ability. The similarly enhanced peak current at Au_nano_/GCE (curve C) attributed to the good conductivity of Au_nano_. After the rGO-Au_nano_ composite modified onto the GCE surface (curve D), the peak current rose further owing to the synergy from rGO and Au_nano_. The electrochemical properties of these modified electrodes were also characterized by EIS.  Figure 3(b) shows nearly straight lines at rGO/GCE (curve B), Au_nano_/GCE (curve C), and rGO-Au_nano_/GCE (curve D), revealing rapid electron transfer between the electrode surface and the electrolyte. Only a small semicircle was observed at bare GCE (curve A), which meant about 100 Ω of electron transfer resistance in this system.

CV technique was also performed to evaluate the electrocatalytic oxidation effect of NADH on different electrodes in 0.1 M PBS (pH 7.2) containing 0.5 mM of NADH at a scan rate of 50 mV·s^−1^. As shown in Figure 3(c), a very small anodic peak was obtained at 0.7 V at the bare GCE (curve A), which meant that the direct oxidation of NADH at the bare electrode was difficult. After the modification of Au_nano_, the anodic peak increased and the working potential negatively shifted to 0.62 V, which might be owing to the good oxidation catalytic ability of Au_nano_ (curve B) [32]. For the rGO/GCE as curve C, a small oxidation peak at 0.24 V was found, showing the excellent catalytic capacity of rGO to NADH and the activation energy of NADH oxidation was declined on the surface of rGO. The reason might be that the presence of abundant defects and edge plane graphite structures in rGO was thought to facilitate the heterogeneous charge transfer at the electrode interface [25, 33, 34]. Thus, the electrons released by NADH could be transferred swiftly with the aid of rGO. After the combination of Au_nano_ and rGO, the oxidation peak current of NADH was similar to that of Au_nano_/GCE, and the peak potential appeared at 0.32 V. The synergic effect of the rGO and Au_nano_ exhibited the capability as a powerful catalyst to the oxidation of NADH [35]. In addition, we also found that no obvious peaks were observed on all these four modified electrodes in PBS without NADH (Figure S1), indicating NADH could be catalytically oxidized with the aid of Au_nano_ and rGO.

The effect of the scan rate on the oxidation current of NADH was also investigated. The CVs of the rGO-Au_nano_/GCE at different scan rates were recorded as Figure 3(d). A good linear relationship of the anodic peak current with the square root of the scan rate in the range of 30 and 100 mV·s^−1^ was presented as the inset of Figure 3(d). The result revealed the oxidation of NADH on the rGO-Au_nano_/GCE was a diffusion-controlling process, owing to the fast electron transfer rate between rGO-Au_nano_ and the electrolyte.

### 3.3. Optimization of the Experimental Parameters

To improve the performance of the NADH sensor, the effect of determination conditions such as the electrodeposition cycles, the applied potential, and the solution pH have been investigated in detail.

**Figure 4:**
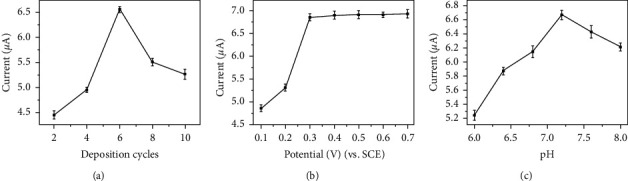
The effect of the catalytic current of 0.5 mM NADH against the (a) number of the electrodeposition circles of rGO-Au_nano_ film, (b) applied potential, and (c) solution pH.

The number of the electrodeposition cycles was optimized by measuring the catalytic current to 0.5 mM NADH using rGO-Au_nano_/GCE with a different rGO-Au_nano_ film. The more electrodeposition cycles exerted, the thicker the rGO-Au_nano_ composite film would be obtained. As shown in Figure 4(a), the oxidation peak current increased sharply as increasing the electrodeposition cycle number from 2 to 6, due to the enhanced conductivity and catalytic activity along with increasing rGO-Au_nano_ deposition. However, the peak current decreased when the number of electrodeposition cycles was more than 6, owing to the fact that the thicker rGO-Au_nano_ film was inclined to drop off. Thus, 6 cycles of electrodeposition of rGO-Au_nano_ deposition were selected in this work.

In order to achieve the best electrocatalytic effect while reducing the overpotential, the applied potential should be optimized. The effect of the applied potential was shown in Figure 4(b). As the applied potential increased from 0.1 to 0.3 V, the peak current increased gradually. Further, increasing the applied potential from 0.3 to 0.7 V led to a relatively constant current value. Therefore, 0.3 V was selected as the optimal applied potential.

The effect of the solution pH on the oxidation of NADH at rGO-Au_nano_/GCE was also studied by monitoring the peak current of NADH in 0.1 M PBS with different pH values from 6.0 to 8.0.  Figure 4(c) shows that the oxidation peak current of NADH increased along with the solution pH value from 6.0 to 7.2, and then decreased. Therefore, pH 7.2 was selected as the optimum for further studies.

### 3.4. Detection of *α*-KG Using GLUD/rGO-Au_nano_/GCE

**Figure 5:**
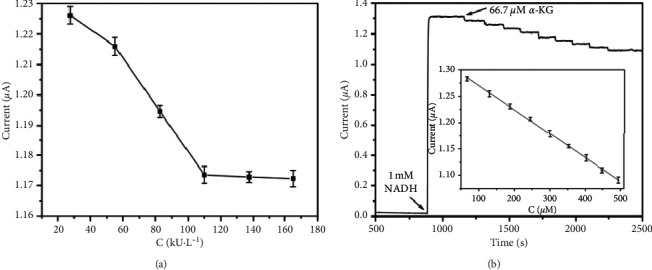
(a) The effect of GLUD concentration on the current response of NADH at GLUD/rGO-Au_nano_/GCE in 0.1 M pH 7.2 PBS containing 312.5 *μ*M of *α*-KG, 1 mM of NADH and 1 mM NH_4_Cl. (b) Amperometric current-time curve recorded at GLUD/rGO-Au_nano_/GCE for the successive additions of 66.7 *μ*M *α*-KG in a stirred pH 7.2 PBS containing 1 mM NADH and 1 mM NH_4_Cl. The applied potential was 0.3 V and the stirring rate was set as 200 rpm.

Based on the catalytic mechanism described in the introduction part, the concentration of *α*-KG could be determined by the depleted amount of NADH at a GLUD/rGO-Au_nano_/GCE. That is to say, the content of *α*-KG was inversely proportional to the catalytic current of NADH. To obtain the optimal performance of the biosensor, the concentration of the immobilized GLUD needed to be investigated further. As seen from Figure 5(a), the current response of NADH decreased quickly along with the GLUD concentration increased from 27.5 to 110 kU·L^−1^, showing a fast transform from *α*-KG to L-glutamate. Then, the current tended to become steady with a further increase in GLUD concentration, owing to the saturation of GLUD loading capacity. From an economic perspective, 110 kU·L^−1^ of GLUD was selected in our experiments.

Figure 5(b) shows a typical amperometric current-time curve recorded at GLUD/rGO-Au_nano_/GCE for the successive additions of 66.7 *μ*M *α*-KG in a stirred pH 7.2 PBS containing 1 mM NADH and 1 mM NH_4_Cl. The response was very fast and the steady-state current response was attained in less than 5 s. The linear response range at GLUD/rGO-Au_nano_/GCE was from 66.7 to 494.5 *μ*M, with the sensitivity of 454 *μ*A M^−1^ and a correlation coefficient of 0.9992. The calculated limit of detection was found to be 9.2 *μ*M (S/N = 3). After the GLUD immobilization, the biosensor sensitivity was decreased due to the enzyme layer act as a barrier that hindered NADH transport. To our knowledge, there are few reports on electrochemical biosensors for the measurement of *α*-KG, and nearly a few references could be compared. As listed in Table 1, our sensing system exhibited a comparable linear range with a bienzymatic flow injection system [37] and a similar detection limit with the other electrochemical biosensors, suggesting the proposed sensor had a good performance in *α*-KG detection.

### 3.5. Reproducibility, Stability, and Selectivity of the Biosensor

**Figure 6:**
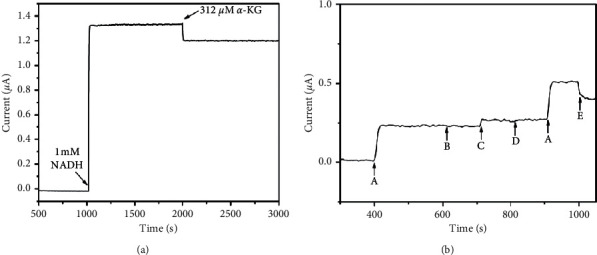
(a) Amperometric current-time curve showing the stability of the *α*-KG response at the GLUD/rGO-Au_nano_/GCE. The first arrow indicates the addition of 1 mM NADH and the second arrow indicates the addition of 312.5 *μ*M *α*-KG. (b) Amperometric current-time curve recorded for the GLUD/rGO-Au_nano_/GCE with the addition of 0.25 mM NADH (A), 0.1 mM of DA (B), AA (C), UA (D), and 312.5 *μ*M *α*-KG (E). The applied potential was 0.3 V. Stirring rate: 200 rpm.

The reproducibility of GLUD/rGO-Au_nano_/GCE was investigated in 0.1 M PBS containing 1 mM NADH and 1 mM NH_4_Cl via recoding the current response during the successive addition of 66.7 *μ*M of *α*-KG. Six sensors were prepared in different batches, and 10 successive measurements were implemented for each electrode, and the RSD of the current response was 4.8 and 4.2%, respectively. The stability of GLUD/rGO-Au_nano_/GCE was also evaluated. The current response for 312.5 *μ*M *α*-KG was measured, and there was nearly no apparent loss during ∼1000 s' operation, as shown in Figure 6(a). Such good stability of GLUD/rGO-Au_nano_/GCE was attributed to the protection of the glutaraldehyde membrane and the biocompatibility of rGO-Au_nano_ for GLUD immobilization.

Selectivity is important for the practical application of biosensors. An assessment of the interference on the amperometric response to NADH was examined at the GLUD/rGO-Au_nano_/GCE in the presence of other oxidizable substances, such as dopamine (DA), ascorbic acid (AA), and uric acid (UA).  Figure 6(b) shows the amperometric responses at GLUD/rGO-Au_nano_/GCE after the addition of various interferents. In the presence of 0.25 mM NADH, 0.1 mM DA or UA could not induce apparent interference, while 0.1 mM AA generated an obvious interference, since AA could be oxidized at lower potential compared with DA and UA [38, 39]. Thus, AA should be removed when the biosensor was used for practical application. One possible method for removing AA was the immobilization of ascorbate oxidase onto the electrode surface [40].

### 3.6. Real Sample Analysis in Human Serum

The designed GLUD/rGO-Au_nano_/GCE was further evaluated in real human serum using the standard addition method. The human serum was diluted 10 times with 0.1 M pH 7.2 PBS. Then, the solution was transferred into the electrochemical cell for analysis. The amperometric current-time measurement was employed for the recovery test to determine *α*-KG in the human serum samples. As listed in Table 2, the recoveries of *α*-KG between 97.9 and 102.4% with RSD values in the range of 3.8 to 4.5% were obtained, indicating the strong potential application prospect in clinical diagnosis.

## 4. Conclusion

In this work, an *α*-KG biosensor based on the rGO-Au_nano_ film was successfully prepared. The rGO-Au_nano_/GCE platform resulted in the improvement of electrocatalytic activity towards the oxidation of NADH with lower working potential, higher sensitivity, stability, and reproducibility. After the modification of GLUD, the electrode exhibited a sensitive response to *α*-KG in PBS via the consumption of NADH, implying good stability of GLUD on biocompatible rGO-Au_nano_ platform and rapid electron transfer ability between electrode and *α*-KG. Moreover, the biosensor was applied to determine *α*-KG in human serum with satisfactory recoveries, illustrating its good selectivity and anti-interference. To summarize, the *α*-KG sensing strategy reported in this work could be expected for real applications in the future.

## Figures and Tables

**Table 1:** Comparison of *α*-KG detection performance with sensors.

*α*-KG sensor	Linear range (*μ*M)	Detection limit (*μ*M)	Ref.
GLUD-Ru/Rh	100–600	20	[8]
GLUD-rMNs-Au_nano_	11.12–52.94	6.25	[36]
GDH-GlOx	0–500	7	[37]
GLUD/rGO-Au_nano_	66.7–494.5	9.2	This work

**Table 2:** Recovery test of *α*-KG in human serum samples (*n* = 3).

Sample	Added (*μ*M)	Found (*μ*M)^a^	Recovery (%)	RSD (%)
1	100	102.4	102.4	4.5
2	200	197.6	98.8	3.8
3	300	293.7	97.9	4.3

^a^Average of three measurements.

## Data Availability

The data that support the findings of this study are available from the corresponding author upon reasonable request.

## Supplementary Materials

Supplementary Materials Figure S1: CVs of different working electrodes in 0.1 M pH 7.2 PBS without NADH at a scan rate of 50 mV·s^−1^.
