# Supplementary material for: Highly Sensitive Amperometric α-Ketoglutarate Biosensor Based on Reduced Graphene Oxide-Gold Nanocomposites
Source: Int J Anal Chem. 2020 Aug 1;2020:4901761. doi: 10.1155/2020/4901761 (PMC7416262; doi:10.1155/2020/4901761)
Supplement: Supplementary Materials — Figure S1: CVs of different working electrodes in 0.1 M pH 7.2 PBS without NADH at a scan rate of 50 mV·s−1. [file 4901761.f1.docx]

Supporting Information

Highly sensitive amperometric α-Ketoglutarate biosensor based on reduced graphene oxide-gold nanocomposites

Gang Peng,^1,4^ Yadong Yu,^1, 3^ Xiaojun Chen^2,^* and He Huang^1,3,^*

^1^ College of Biotechnology and Pharmaceutical Engineering, Nanjing Tech University, Nanjing 211800, PR China

^2^ College of Chemistry and Molecular Engineering, Nanjing Tech University, Nanjing 211800, PR China

^3^ College of Food Science and Pharmaceutical Engineering, Nanjing Normal University, Nanjing 210023, PR China

^4^ College of Food Engineering, Anhui Science and Technology University, Fengyang 233100, PR China

* Correspondence: chenxj@njtech.edu.cn (XJ Chen); huangh@njtech.edu.cn (H Huang)


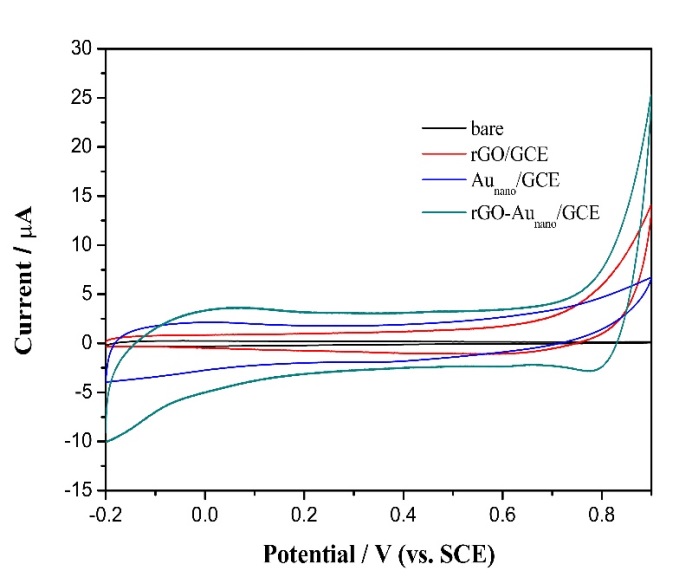


Figure S1. CVs of different working electrodes in 0.1 M pH 7.2 PBS without NADH at a scan rate of 50 mV s^-1^.
